# Controlled Electromagnetic Field Based Safety System for Handheld Circular Saw

**DOI:** 10.3390/s22228593

**Published:** 2022-11-08

**Authors:** Pedro Teixidó, José M. Hinojo-Montero, Juan Antonio Gómez-Galán, Fernando Muñoz-Chavero, Trinidad Sánchez-Rodríguez, Juan Aponte

**Affiliations:** 1 OnTech Security LLC, C/Hispano Aviación, 7-9, 41300 La Rinconada, Seville, Spain; 2 Department of Electronic Engineering, University of Seville, 41092 Seville, Spain; 3 Department of Electronic Engineering, Computers, and Automation, University of Huelva, 21007 Huelva, Spain

**Keywords:** power tools, circular saw, safety, health, human detection, tele-care

## Abstract

This paper presents the design of a safety system based on controlled electromagnetic field (CEMF) sensing technology to prevent accidents caused by power tools, especially related to handheld circular saws. The safety system creates an invisible protection bubble of electromagnetic field around the cutting edge. The system can provide early warning or critical warning when a person penetrates the safety bubble. This paper covers how the CEMF technology has been adapted to add value within this application where it needs to coexist with a difficult environment of metallic parts turning thousands of times per minute, strong vibrations, and different ranges of materials to be processed. The proposed contactless solution successfully detects the user, providing enough time for the power tool to totally stop its movement before touching and harming the user. This key property has required a careful optimization of the electromagnetic field generation, the design of a shield circuitry capable of operating properly in a large metal device, and the development of a multi-frame algorithm to address the stringent requirements related to the ability of the system to react to both very fast and very slow events. The feasibility of the system has been validated by a virtual testbench.

## 1. Introduction

The identification of information regarding objects that are physically close to each other, without requiring physical contact, is a key task known as proximity detection. This information, which can be obtained by means of proximity sensors, is required by a variety of electronic platforms and industrial equipment. As a result, proximity sensors are a critical element in a wide range of systems [1,2,3,4]. The most commonly used technologies to provide proximity detection are optical, ultrasonic, inductive, and capacitive, and are usually based on the measurement of a change in either an electrostatic field, or an electromagnetic field [5,6,7,8,9,10]. For inductive sensing, the detection is limited to conductive and/or ferromagnetic objects, and the maximum sensing range is typically similar to the coil’s diameter. Optical and ultrasonic technologies can detect both conductive and non-conductive objects, but the implementation of a complex light source or sound wave actuator can be complicated, especially when applied to detecting/interacting with fabrics. Furthermore, an object’s surface polish, as well as its material quality, can affect the detection range.

Capacitive proximity sensors are capable of detecting both conductive and non-conductive objects, which makes them suitable to detect objects, as well as humans [11,12,13,14,15]. The sensing technique is easy to set up, and fewer components are required for its implementation, compared to other technologies. Furthermore, its working mechanism makes it the most suited for printed implementations, since it only needs a conductive electrode as the sensing element, regardless of its shape. While human-sensing includes several disciplines, the focus of this work is on the challenge of instantaneous sensing at the lowest level, with the sensing mechanism being based on the interaction of a human with an electric field, as they approach an electrode which is part of the capacitive sensor front end.

A wide range of industrial applications have been using capacitive proximity sensors for decades, due to the distinct advantages they provide, in terms of affordable construction costs, low energy consumption, a broad range of monitoring capabilities, outstanding dynamic response, and a structural design that is both adjustable and flexible, compared to other proximity detection technologies [16]. In addition, during the past few years, capacitive proximity technologies have continued to gain significant attention from researchers, who aim to further enhance their performance regarding sensitivity, resolution, stability, thermal drift, compactness, as well as the power efficiency of the required electronics [17,18]. The fundamental principles of the technology, as well as the trade-offs regarding sensitivity, resolution, and signal strength, are crucial elements that allow for a good capacitive proximity sensor design. Since the structure of the sensor can have an impact on the measurement, optimizing the parameters of the electrodes for the highest design priority is also an element of importance in a practical application.

In this paper, we report on the development and evaluation of a human detection system based on the CEMF sensing technology that is well suited for the safety of an operator handling a circular saw. In today’s market, it is possible to find power tools already capable of stopping in case of danger, but the detection of danger situations happens on contact with the cutting element. Therefore, even though minor tissue damage occurs, it is undesirable that the protection starts after the damage has occurred. It should always be the other way around, and here is where CEMF sensing technology can make the difference, as it allows projection and control of the electromagnetic field around the blade, generating an invisible safety bubble whose reading will provide early notice of any potentially dangerous situation. CEMF technology is based on the generation, measurement, and control of electromagnetic fields. The evolution of this physical phenomenon has served for developing a technology that continuously measures small disruptions in the field lines, detecting the presence of bodies or objects. CEMF technology is based on capacitive sensing. Among capacitive sensors, oscillator based sensors are used due to the high sensitivity in frequency due to variations in the capacitance to be measured, and the frequency stability in case of different disturbances, such as vibrations, temperature drifts, supply voltage deviations, etc. A driving electrode coupled to an oscillator circuit is used to provide an electrical stimulation. The human hand approaching the electrode distorts the electric field. The resulting change of the self-capacitance between the transmitter electrode and the distant ground is measured.

As previously mentioned, the key improvement of the proposed solution over current alternatives is the ability to react before any damage has occurred, but there are other important advantages such as reducing the mechanical stress in emergency stop operations due to the longer available time. This will surely lead to a reduced number of failures and a longer tool life. Additionally, thanks to the ability of CEMF technology to acquire and classify field distortions, it is possible to provide an early warning to make the user aware of the endangering situation, and perhaps correct it and avoid an emergency stop. In the professional market, such a warning notice could be logged and analyzed to determine if certain employees are too often putting themselves in danger and could make good use of any corrective action such as safety training or relocation to a different department.

This paper presents how an innovative and disruptive safety mechanism for circular saws can be turned into reality using the CEMF sensing technology. Thanks to this unique technology and to the study of how to apply it to a circular saw, it is possible to achieve the fine sensitivity, the fast reaction time, and the reliability required for such an application, to distinguish regular operation from dangerous events in order to stop the power tool in time and avoid any unnecessary harm that could have otherwise occurred. Throughout this paper, the safety system specifications are presented as an entry point before moving forward to the feasibility analysis, architecture definition, design, physical implementation, and validation, finalizing the paper with the conclusions.

## 2. Feasibility Analysis

**Figure 1:**
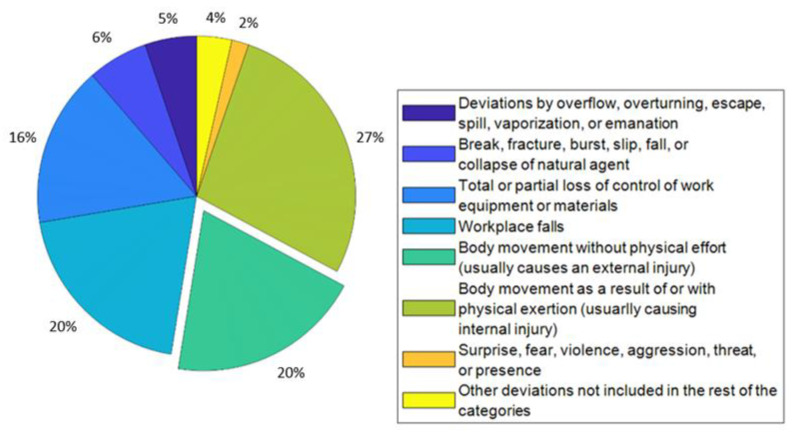
Accidents at work with illness leave of injured employees.

According to the report of status of health and safety at work released [19] by the Spanish National Institute of Health and Safety at Work, 20% of accidents occur by the user moving towards the tool (see Figure 1).

The approaching speed of the body is considered by safety certification entities as 2 m/s. According to [20], a brushless motor can be stopped in 83 ms. In addition, the main players of the power tools market believe that it is possible to reach a stopping time of 25 ms, but it would have a significant impact in pricing. For this reason, this paper will consider 75 ms as the nominal stop time. Based on the approaching speed and the stop time, it is possible to calculate the detection distance: *Target Distance = Approaching Speed* × *Stop Time*, yielding 15 cm (2 m/s × 0.075 s). As the acquisition and processing are not immediate, the system needs to be sensitive at a farther range than the target distance. The next step is to estimate the required CEMF range.

CEMF technology provides a strong field generation and a great sensitivity. However, because of the metallic environment an initial characterization needs to be carried out to guarantee that there will be enough sensitivity to satisfy the 15 cm target.

Although final electrode size, shape, and location remain unknown, an electrode size of 30 × 12 mm has been defined. This small size is considered to be worst-case scenario in terms of sensitivity.

**Figure 2:**
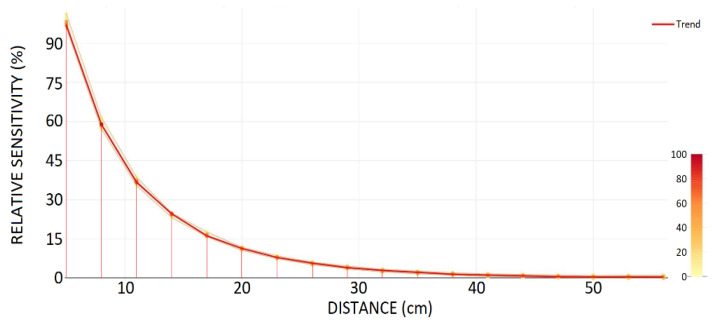
Electrode sensitivity for worst-case electrode size (30 × 12 mm).

Figure 2 shows CEMF signal change vs. distance ranging from 5 to 56 cm. Note how the sensor starts detecting the plate between 40 cm and 50 cm, and it is clearly visible at distances below 25 cm. This proves that the field penetration and sensitivity range are enough for this application.

**Figure 3:**
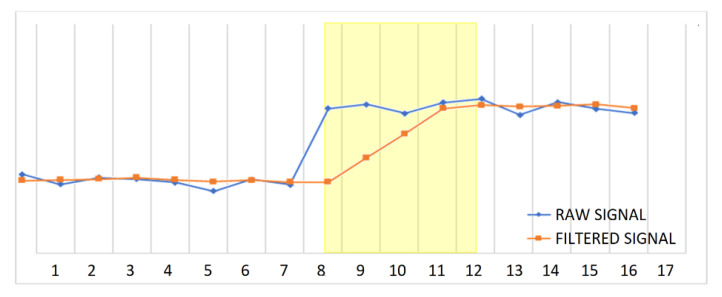
Filter delay.

The next step into this feasibility study is to verify the acquisition speed. As the CEMF signal will suffer large noise due to mechanical vibrations of surrounding mechanical parts, the usage of a filter will certainly be required. The chosen filter will delay the acquisition output by 4 samples (See yellow area in Figure 3).

As seen previously, there is around 25 cm of detection range, and the target distance is 15 cm. So, there is 10 cm of spare distance that could be used for acquisition and processing. Using the approaching speed of 2 m/s, this 10 cm will translate to a 50 millisecond budget. After the first sample has been fed into the filter, 3 new samples are required for the event to achieve its full amplitude at the filter’s output. Therefore, the sample time is 16 ms.

**Figure 4:**
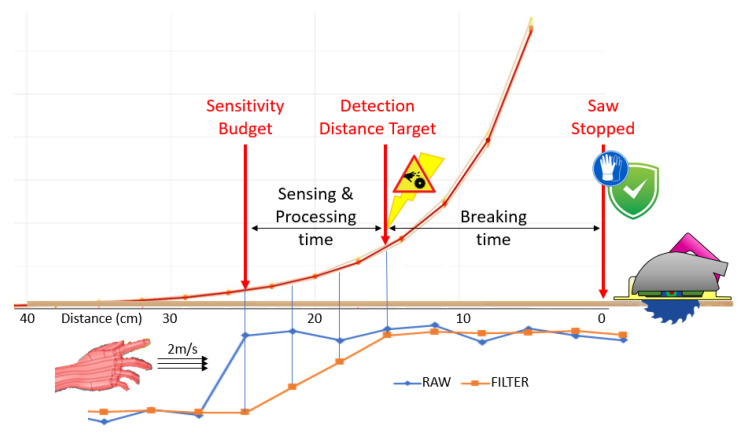
Sensitivity budget diagram.

Figure 4 synthetizes all relevant information used throughout this feasibility analysis. It can be seen clearly that, as the hand approaches the saw at 2 m/s, it is detected for the first time by the CEMF technology at a distance of 25 cm from the saw. Three samples (or 10 cm) later, the signal will be fully visible at the output of the filter, and the alarm will be sent to the control of the tool. At this point, the power tool will perform an emergency break, stopping the motor in 75 ms (or 15 cm), which is the current distance between the hand and the saw. This means that, should the impact take place, the saw will be totally stopped by the time it happens.

## 3. Architecture Definition

Once this work matures enough to be integrated in a product designed for the consumer market, the architecture must be autonomous, yet simple, reliable, and affordable. However, at this stage this is no more than a research project where the boundaries of the CEMF technology will be tested to discover whether or not it suits the requirements to dramatically improve safety in power tools where metal rotating elements are constantly spinning and propagating vibrations all over the product’s body. Therefore, the focus will be on how to integrate the CEMF technology in the tool rather than on the compactness of the electronic design.

**Figure 5:**
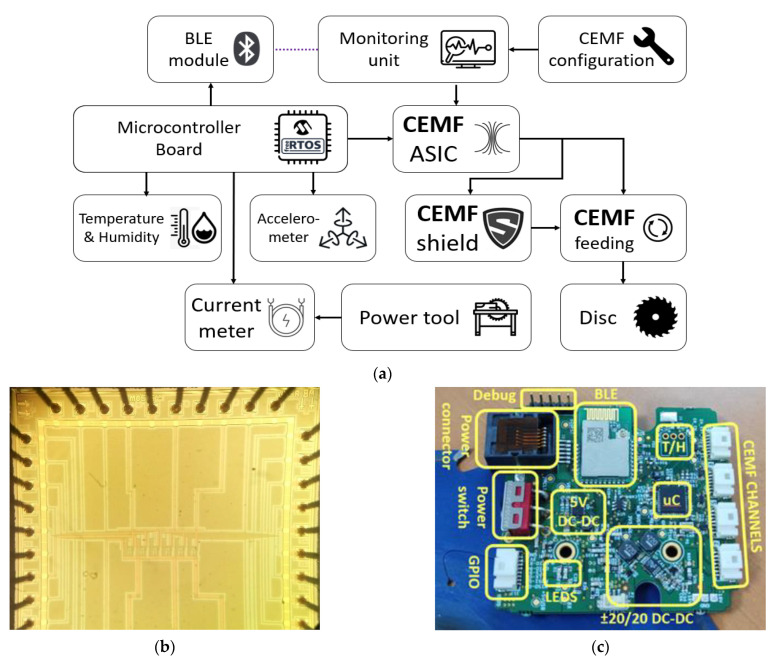
(**a**) Architecture diagram. (**b**) CEMF ASIC microphotograph. (**c**) Microcontroller board.

Now a clear goal is set, the architecture used for the work is shown in Figure 5a. The CEMF sensor has been integrated in an application specific integrated circuit (ASIC) via MOSIS service in AMI 0.5 µm CMOS technology as shown in Figure 5b. The power tool is connected to a monitoring unit through Bluetooth low energy (BLE), which will allow for an accurate and real time monitoring of the internal CEMF data without compromising the system ground due to electrostatic charge possibly accumulating due to the friction of the moving parts. Figure 5c shows the microcontroller board used. It is based on a Microchip PIC24 running free-RTOS to recycle previous knowledge and already built firmware libraries. The board includes an onboard temperature and humidity sensor that will provide internal readings for environment compensation, as well as an accelerometer and a current sensor that will provide information about the status of the tool, yet these two sensors should not be required in production, as the tool status could be directly retrieved from the tool control unit instead of estimating it from the measurements of the vibrations and the power consumption of the motor. The CEMF ASIC used will generate the CEMF signal to be injected into the sawing disc through a coupling mechanism. Finally, a CEMF shield signal will be generated to protect the sensitive signal towards where the sensitive field is generated, and thus confine the sensitive area within the element of interest, which is the blade itself.

## 4. Design

### 4.1. Initial CEMF Integration

As stated previously, the focus of this paper is on how to adapt the CEMF technology to a power tool and, for this reason, we will pay special attention to the mechanical aspects rather than to the electronic design. There have been several implementations and simulations before reaching the current configuration.

Originally, a classic approach was taken by positioning the electrode in front of the cutting edge of the disc. No simulations were carried out at this stage, but it was an initial approach to start feeling how the CEMF technology would perform in such an environment. As the baseplate of the saw used was a large, grounded metal plate, the electrode could not be installed on it directly, as it would significantly damage the sensitivity. Therefore, a plastic separator was designed to provide some separation from the baseplate.

**Figure 6:**
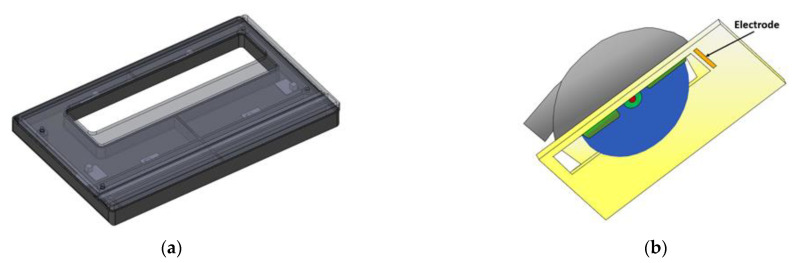
(**a**) Baseplate insulator, (**b**) electrode position.

**Figure 7:**
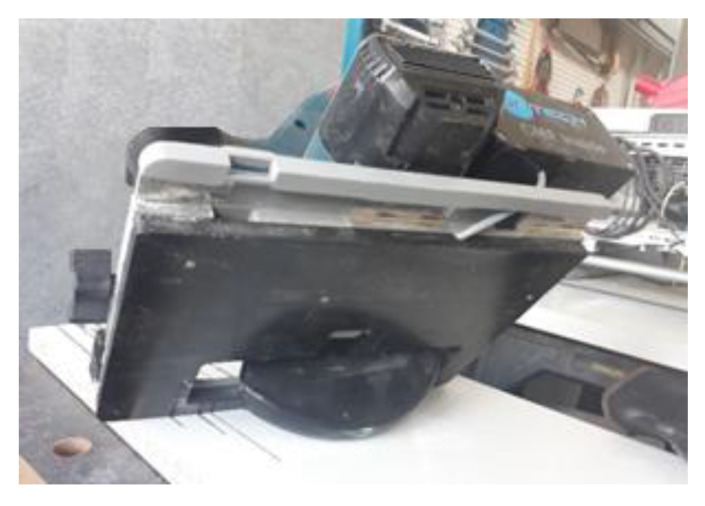
Initial prototype with baseplate insulator.

The electrode was then installed on the inner side of the plastic insulator as shown in Figure 6; this way, it was protected from being damaged due to contact with the material being cut. Figure 7 shows the initial prototype with baseplate insulator.

This initial approach worked correctly when the object approached the front of the saw and it was in a short range of the electrode (I.E: ~5 cm), but it clearly failed when the hand approached the back of the disc or the bottom side of it. Despite being far from what is intended in this paper, this initial approach showed, for the first time, the CEMF technology operating in a working circular saw, and it also showed that in order to achieve a more or less homogenous protection around the disc, the electrode must be the disc itself. The new approach presented in this paper will provide a more constant range of protection among all disc blades. After concluding this short experiment to obtain a feeling of the CEMF technology operation for this application and some initial insights, it was time to carry out some electromagnetic field simulations to verify the range and shape of the field that will act as the invisible shield for the user.

### 4.2. CEMF Field Simulations

As introduced in the last paragraph, the disc will be used as the electrode. So, even though the shape, size, and position of the electrode are all predefined, there are other elements that can be used to tweak and improve the sensitivity of the system. A 3D multi-physics simulator (ANSYS HFSS) has been used to optimize the electromagnetic field generation in order to achieve the 25 cm target coverage.

**Figure 8:**
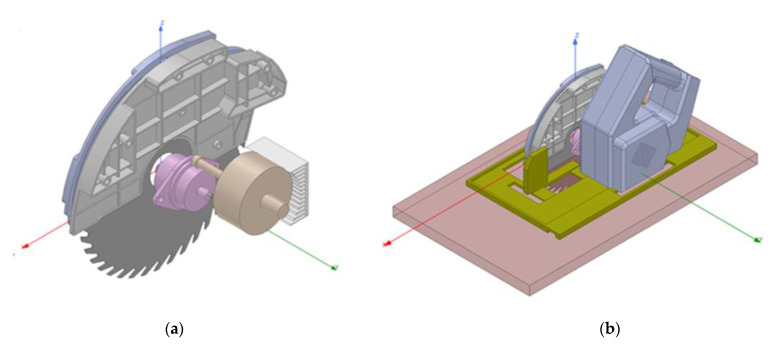
(**a**) Internals of the tool. (**b**) Full tool model.

The first part modeled in ANSYS was the tool itself. In order to provide more confidence to the simulations, it was required to model all the internal elements as close as possible to the real tool as shown in Figure 8. After modeling the parts with SOLIDWORKS, they had to be adapted for ANSYS solver to handle them properly. After that, materials were assigned to each part so that the electromagnetic simulation could accurately calculate the influence among the fields. 

**Figure 9:**
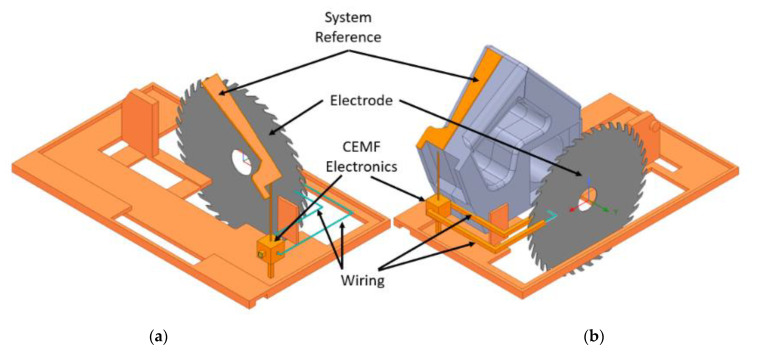
(**a**) CEMF components: exposed coaxial core, (**b**) CEMF components.

The second step was to install on the model the CEMF system to generate the fields. Due to the nature of this technology, it is required to model each of the required parts precisely. In Figure 9, the electronic circuit, the sensing electrode, the system reference, and the wiring can be appreciated. The orange parts are connected to system ground. The CEMF electronics are also encased within a grounded box. The CEMF signal is generated within this grounded enclosure and conducted through the coaxial cables into the saw blade. A system reference pad propagates the ground to the handle of the tool. This is intended to couple to the user and provide a solid reference to generate a larger electromagnetic field.

**Figure 10:**
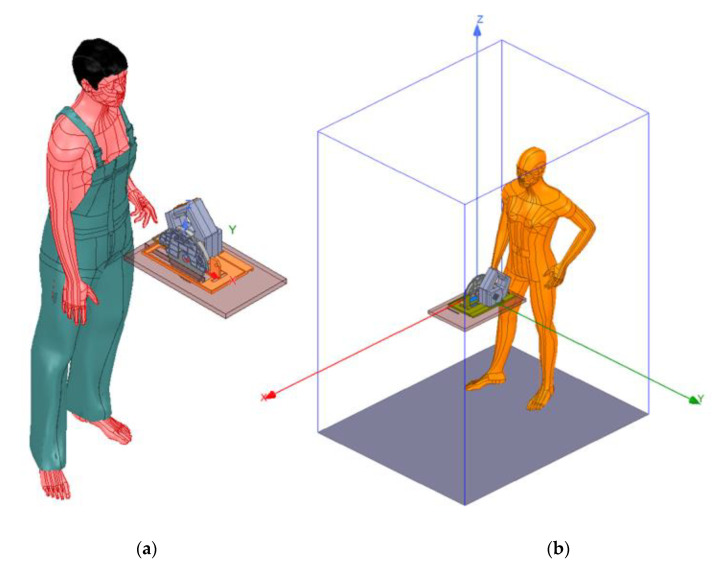
(**a**) User model, (**b**) simulation box.

The next step was to model the world around the tool. That is, the user, the wood, as well as the space where everything happens, also called a simulation box as shown in Figure 10.

**Figure 11:**
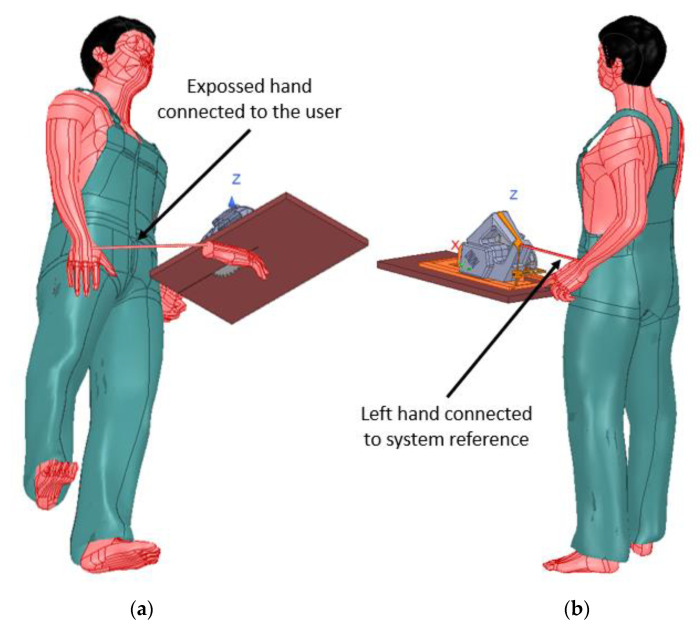
User interacting with the circular saw: (**a**) exposed hand, (**b**) system reference hand.

The user has been modeled through a simple approximation, assuming it is made of salty water. It is a simple but effective model. The simulation box assumes a floor made out of a non-conductive material such as concrete. As the human model is rigid, an extra hand has been modeled and connected to the user. This hand will be used to simulate the exposed hand being in danger. A similar thing happens with the handle, as the human model used cannot grab the handle, so the system reference has been connected to its left hand as shown in Figure 11.

The simulations for this second design showed low performance. Therefore, a third iteration was carried out to extend the range and satisfy the requirements. Before presenting the simulation results to the reader, the improvement made on the third iteration will be introduced here. This will allow us to expose the results of both designs as a comparison between the two approaches in a much more interesting way, showing the field of both solutions side by side.

**Figure 12:**
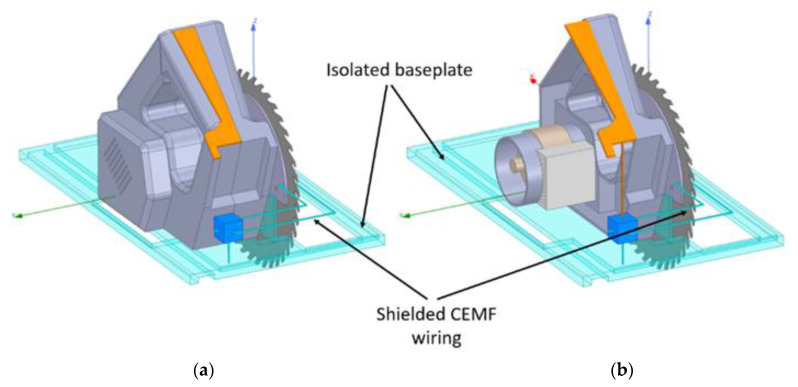
(**a**) Modeled CEMF components v3: case. (**b**) Modeled CEMF components v3: no case.

The third design, which allowed us to improve the range over the 25 cm target distance, introduced two changes. The idea behind it is to control the field by minimizing the coupling to the metal body of the tool and maximizing the coupling to the environment surrounding the tool. In order to do so, the CEMF signal is protected with an active shield along its path to the blade, and the second change isolated the baseplate from ground. This new design is shown in Figure 12 and maintains the system reference pad to couple to the user.

Note the shielded wiring and the isolated baseplate colored in light blue vs. orange in Figure 12. This is because the CEMF wiring is no longer shielded with ground, and the baseplate is left floating to avoid it sucking a large part of the field. Therefore, ground is only populated on the system reference pad still colored in orange.

**Figure 13:**
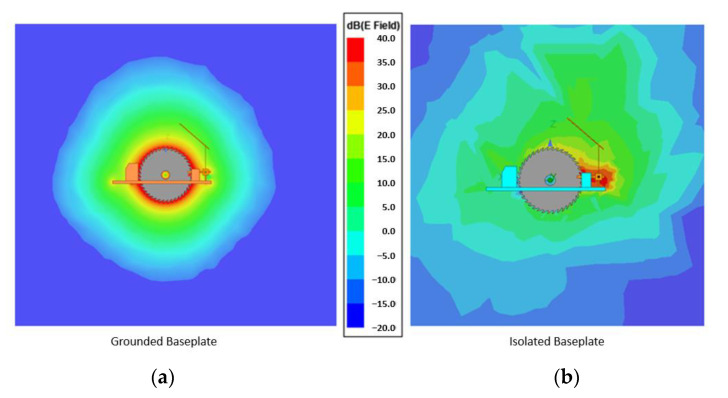
(**a**) Field simulation with saw only (without the user nearby): grounded backplate, (**b**) field simulation with saw only (without the user nearby): isolated backplate.

Despite being a small change in the model, it represents a big improvement in terms of sensitivity. The first simulation in Figure 13 shows how the field is generated when no body part is near the blade. The field around the solution with the grounded baseplate is more homogeneous, but it is kept tight around the tool. Meanwhile, the solution with an isolated baseplate has a much larger field, meaning that it will be able to detect from afar. All plots represented here show the field in V/m displayed in logarithmic scale (dB). The magnitudes are the result of injecting CEMF signal in the ports. This means around ±10~20 V and 100~900 kHz.

**Figure 14:**
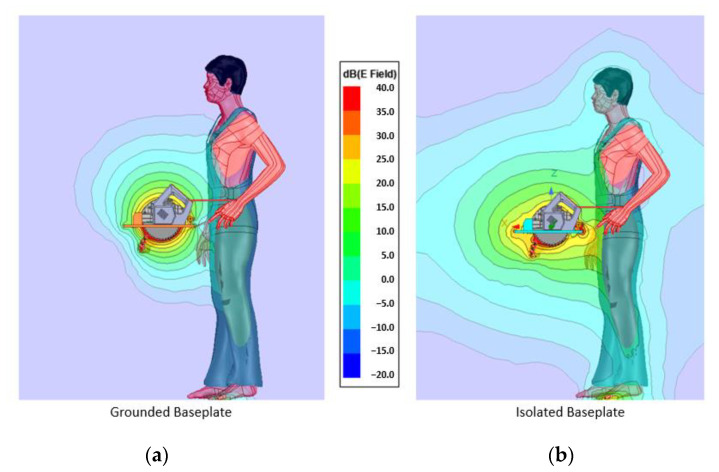
(**a**) Simulation with user (overview): grounded backplate, (**b**) simulation with user (overview): isolated backplate.

The second simulation is with the user near the blade as shown in Figure 14. This will allow observation of how the field diverts once a body part is in range. As in the previous simulation, the field on the shielded implementation expands more. It even goes beyond 50 cm.

**Figure 15:**
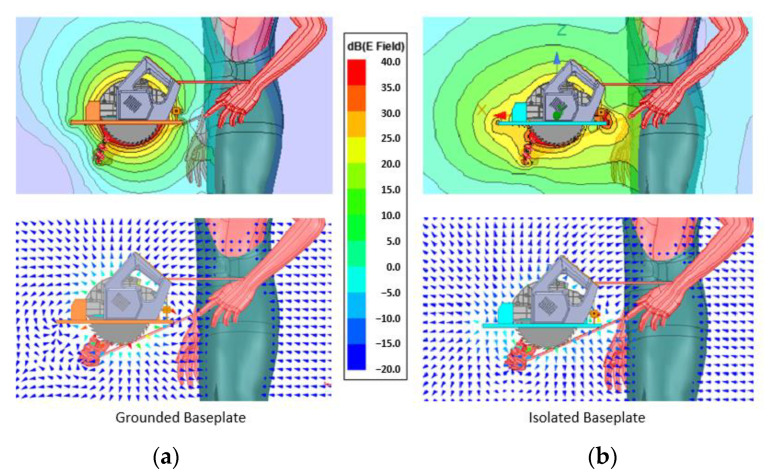
(**a**) Field distribution around the exposed hand: grounded backplate, (**b**) field distribution around the exposed hand: isolated backplate.

**Figure 16:**
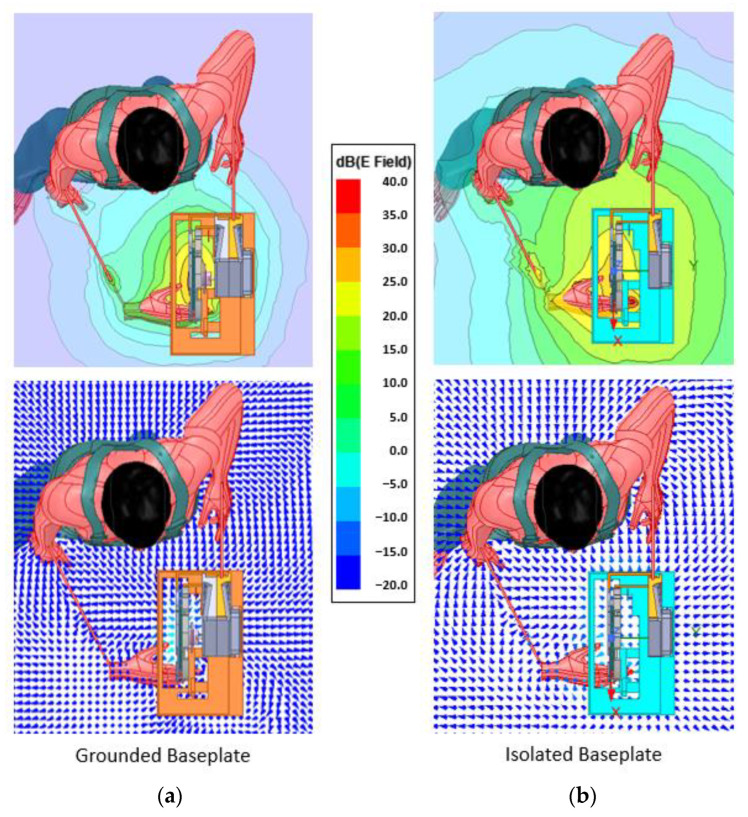
(**a**) Field distribution around the exposed hand: grounded baseplate, (**b**) field distribution around the exposed hand: isolated baseplate.

Now let us focus on the exposed hand itself to understand how the field closes around the human body as shown in Figure 15 and Figure 16.

The field is tighter around the grounded solution, and even though it wraps around the hand, it hardly reaches 10 cm. The isolated version, as observed in previous simulations, generates a wider field, being able to detect the hand from further away than the required 25 cm.

**Figure 17:**
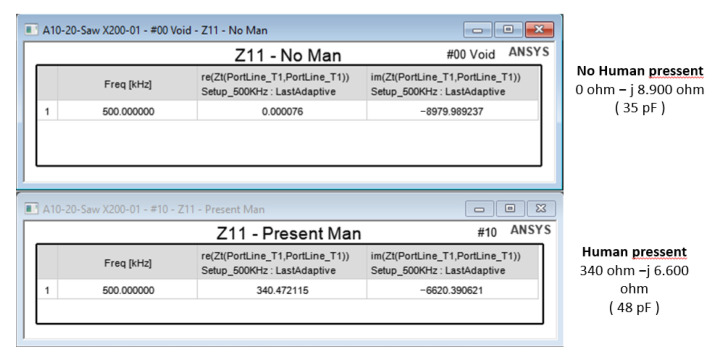
Complex impedance at the point of interest for isolated baseplate solution.

Throughout all simulations, the isolated baseplate approach provides larger range, thus, it clear that it is the correct choice for this work. For this reason, it has been used to calculate the complex impedance at the point of interest with and without a human (modeled by assigning the permittivity and conductivity values of salty water). The difference between these two figures (Figure 17) enables the detection of the event.

Through the simulations, it has been proven that the required range is achievable; the only requirements are: (a) isolate the baseplate; (b) protect the CEMF sensitive signal with a strong shield signal. For this last requirement, the generation of a precise shield signal strong enough to operate in a large metal device is required. If this signal is achieved, the resulting solution also provides a bonus: as it uses the blade itself to generate the electromagnetic field, this solution would adapt seamlessly to different blade diameters, ranging from 15 cm up to 23.5 cm, maintaining a similar sensitivity regardless of the diameter of the blade used. Previously, as the electrode was installed in the baseplate, the sensitivity of the protection mechanism when working with a 23.5 cm disc was reduced by 8.5 cm compared to when the saw was operating with a 15 cm disc.

### 4.3. Shield Implementation

**Figure 18:**
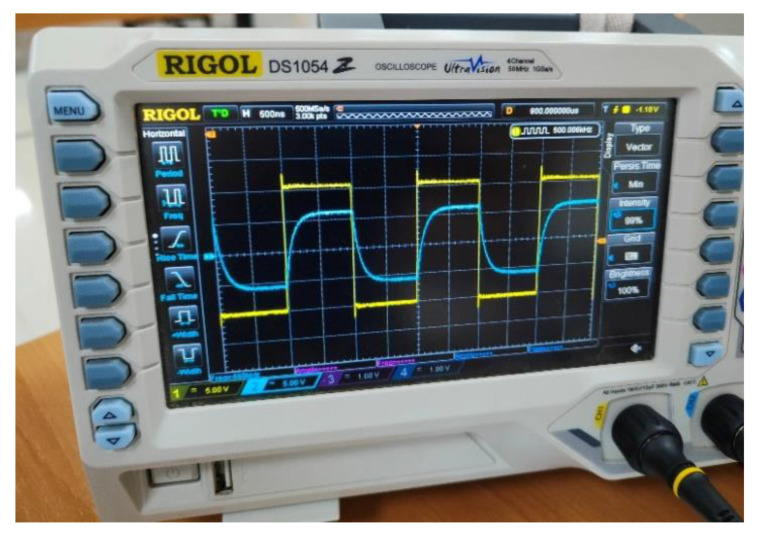
Shield signal using initial follower.

Originally, the standard shield generation was used. This implementation uses a follower to replicate the CEMF signal. However, when it was tested on the power tool the circuit struggled to operate properly. As shown in Figure 18, the shield signal (blue) cannot follow the original CEMF signal (yellow). Note how the shield signal suffers from non-linearities and low amplitude because of the impedance offered by the tool being way out of the operating margins of the standard implementation.

**Figure 19:**
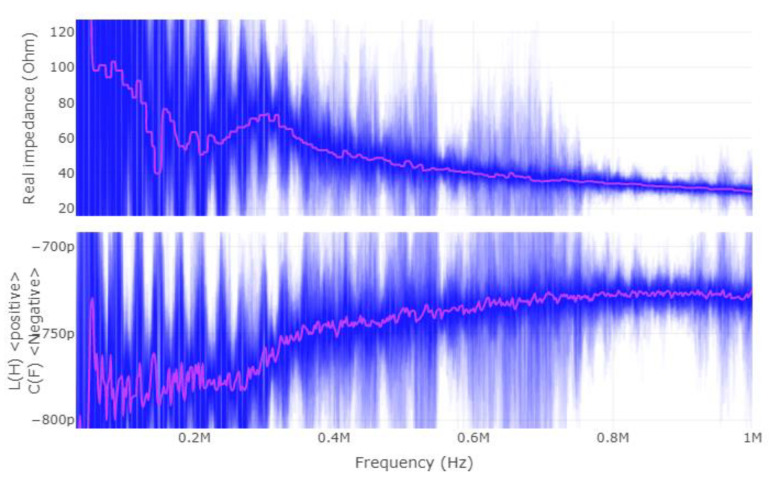
Shield–ground complex impedance (device suspended in the air).

**Figure 20:**
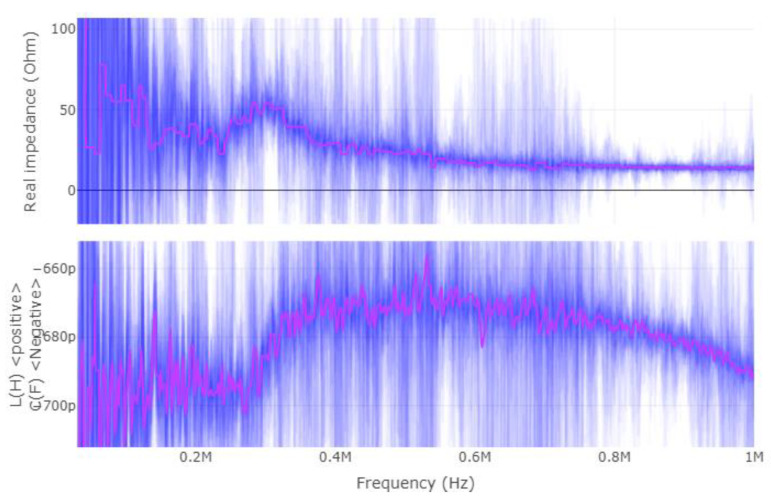
Shield–ground complex impedance on dry wood.

**Figure 21:**
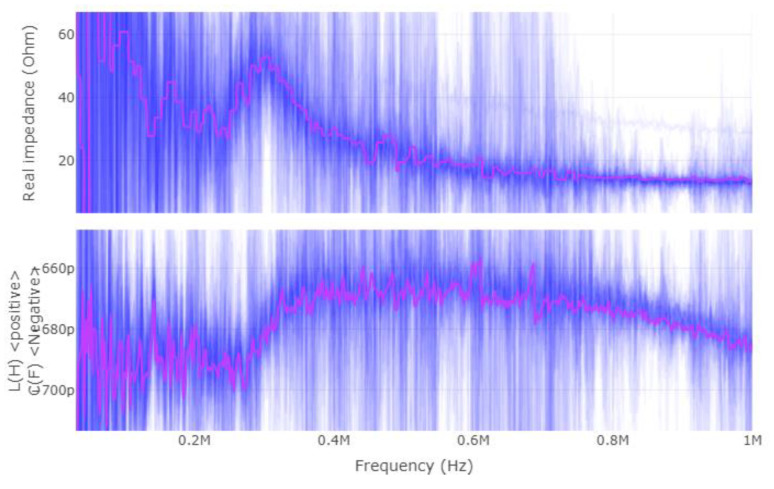
Shield–ground complex impedance on wet wood.

Once more, this application requires a different way of applying the CEMF technology to it. Before designing a new shield circuit, a deeper analysis of what is required will be carried out. Considering that the CEMF amplitude and frequency are fixed at around ±10~20 V and 100~900 kHz, respectively, the most critical parameter is the complex impedance presented by the load. For this reason, the complex impedance of the tool was measured with a vector network analyzer (VNA) as shown in Figure 19, Figure 20 and Figure 21 (Blue represents the distribution of the complex impedance, and red line displays the median value for each frequency step).

The impedance of the shield to ground is in the range of 20~50 Ω and 680~780 pF across all three evaluated scenarios. By running some simulations, the new shield circuit should provide 97 mA of current, and have a slew-rate of at least 604.10 V/µs, as well as be able to handle +/−20 V on both input and output pins.

**Figure 22:**
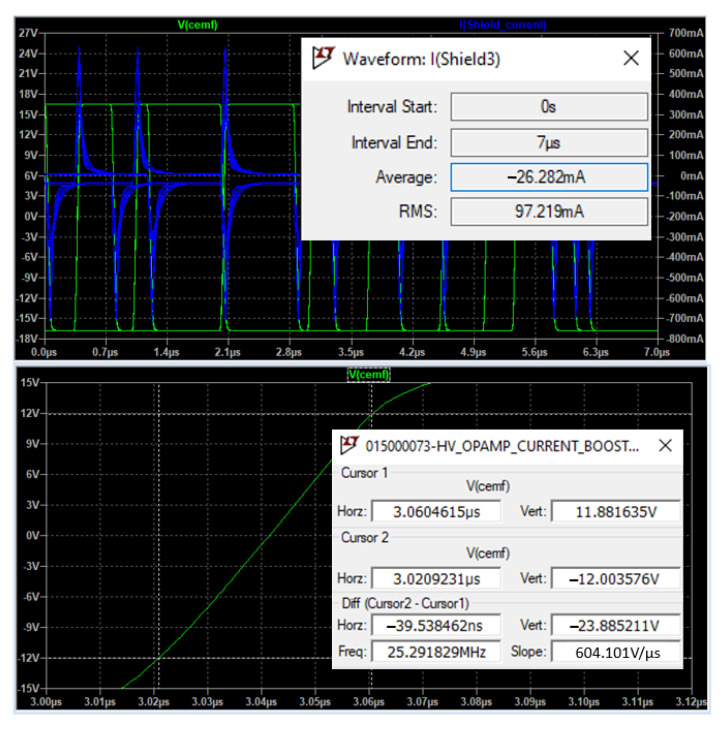
Voltage/current simulation of an ideal shield.

Figure 22 shows the current required by an ideal shield, and has been simulated through 27 combinations resulting from iterations over the following 3 parameters:

- Frequency: 400 kHz, 500 kHz, and 600 kHz.
- Resistance: 20 Ω, 50 Ω, and 70 Ω.
- Capacitance: 650 pF, 750 pF, and 850 pF.

The RMS current is, as mentioned previously, 97 mA, and this is precisely the most critical parameter that has been considered through the design of a discrete solution capable of delivering good performance while connected to the power tool.

**Figure 23:**
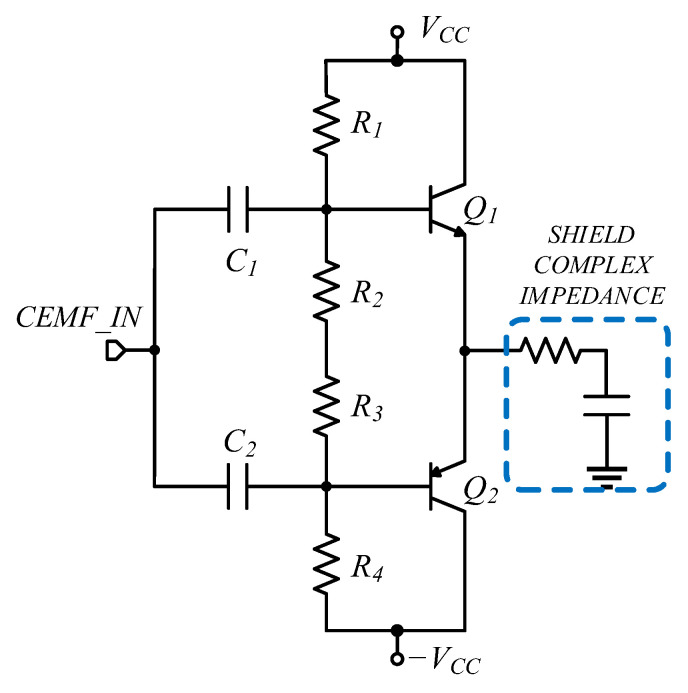
Discrete shield design solution.

**Figure 24:**
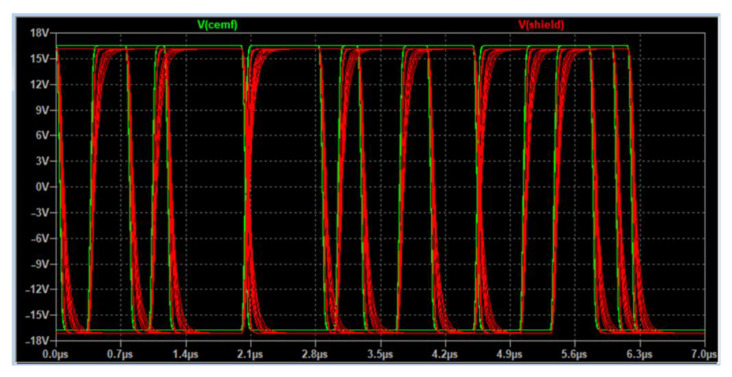
Discrete shield input and output signals.

Figure 23 shows the circuit solution for the discrete shield providing a proper performance as is illustrated in Figure 24.

**Figure 25:**
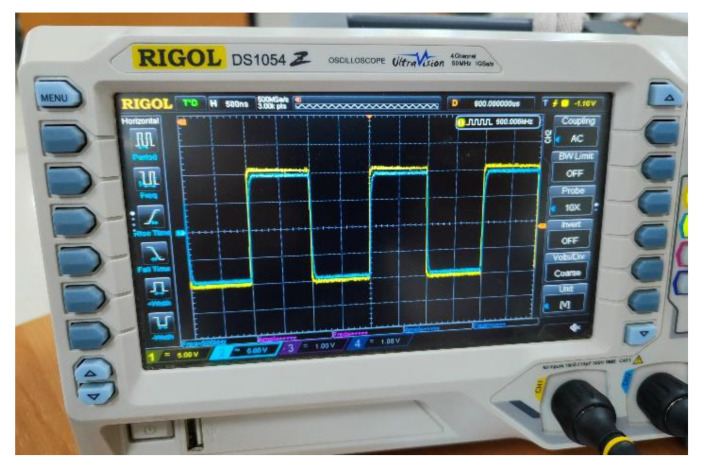
New shield signal connected to the power tool.

Figure 25 shows how the implementation designed performs properly across the different parametric simulations, and the shield signal (blue) can follow the original CEMF signal (yellow). The next step was to assemble it and verify that the issue displayed in Figure 18 had vanished with this new implementation.

After assembling the new design and connecting it to the power tool, it successfully copied the CEMF signal into the shielded wiring, with no apparent distortion.

## 5. Detection Algorithm

**Figure 26:**
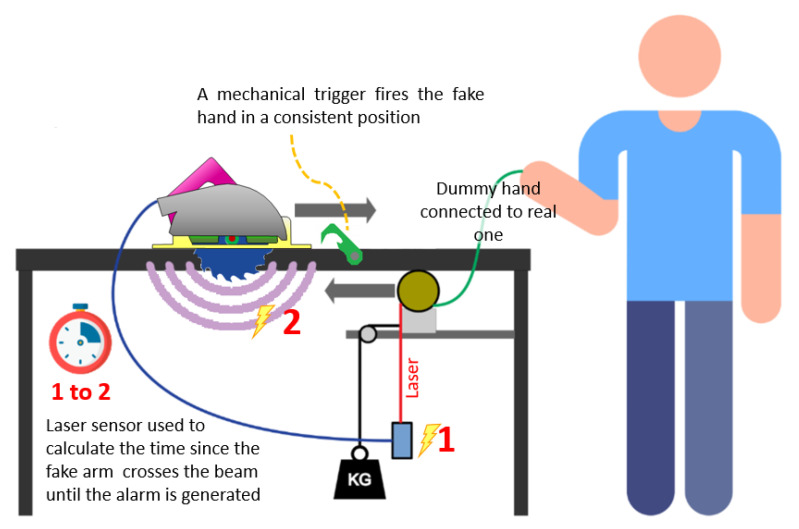
Test platform diagram.

The first step to implement any algorithm is to understand what the data look like and how they relate to the events of interest. For this reason, data collection and labeling were carried out while cutting different pieces of wood and emulating danger situations by using a fake hand/arm with a jig that allowed us to reproduce the approaches in a consistent manner. Figure 26 shows the test platform diagram.

**Figure 27:**
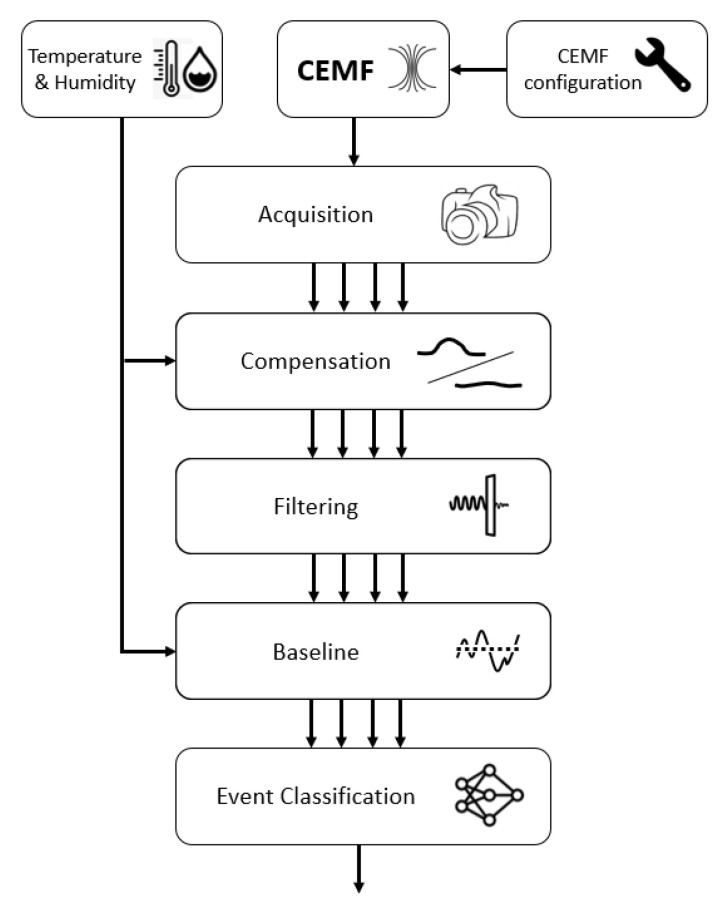
Algorithm overview diagram.

Figure 27 shows a diagram of the algorithm implemented. First of all, the CEMF is acquired at multiple sample rates according to the configuration parameters stored in the NVM. The acquired CEMF signal is them compensated by using the CEMF data themselves and environmental information. Once compensated, it is pushed through a filter that will remove unwanted noise. The filter data will then be zeroed thanks to a tracking baseline. At this point, the data are ready for the classifier to spot dangerous situations and send the stopping signal to the control of the tool if required.

### 5.1. Compensation

**Figure 28:**
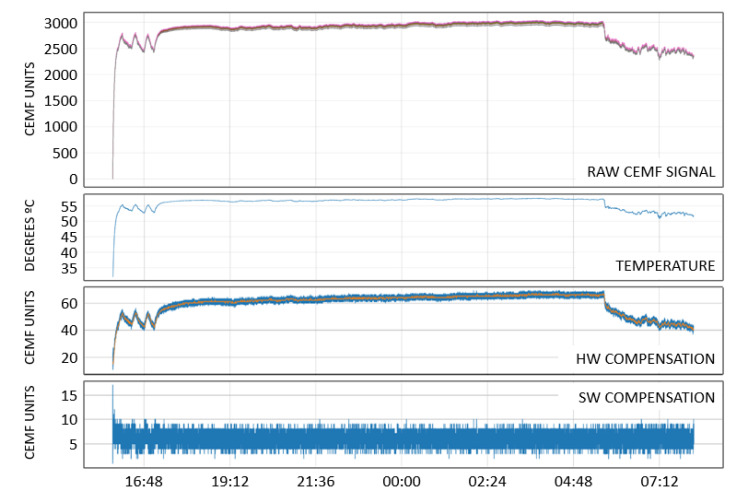
Data compensation mechanism.

Temperature has also been acquired to remove undesired environment effects from the signal. After training different adaptive compensation algorithms using different data windows, it has become evident that the most sensitive choice for this work is to use a linear compensation. The reason for it is that this application requires an instantaneous response. Temperature changes have a good mapping into CEMF signal changes, but due to the nature of the application there is some mismatch between temperature and signal that cannot be eliminated through a linear compensation (HW compensation), therefore a second stage was carried out called SW compensation consisting of absorbing slow and fast variation into a tracking baseline. Then, this baseline was removed from the result of the HW compensation. Figure 28 shows a 12 h period captured where the compensation was able to eliminate a large signal swing of around 3000 CEMF units down to 5 units. Note that the different colors on “RAW CEMF SIGNAL” plot are each one of the channels, and the orange line on “HW COMPENSATION” plot is the mean of the compensated signal.

### 5.2. Multi-Framerate

In order to respond as fast as possible, a high framerate might seem ideal for this application. However, the truth is that a high framerate has the risk of interpreting slow or very slow approaches as environmental changes. For this reason, it was decided to create 4 instances of the algorithm, each one of them targeting different approach speeds. These 4 instances were, obviously, configured with different parameters. This allows for the detection of both slow and fast approaches seamlessly.

**Figure 29:**
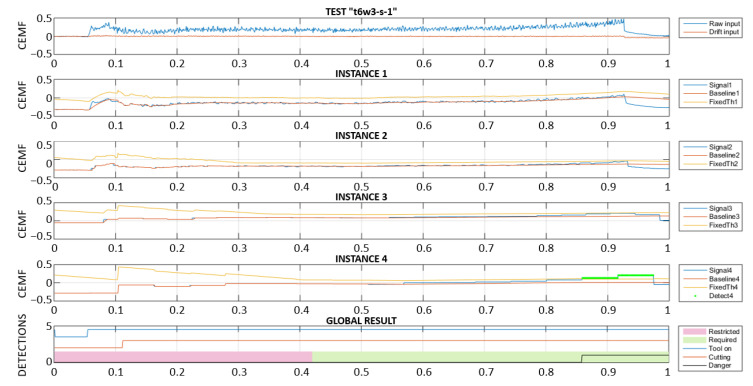
Multi-framerate algorithm: slow approach detection.

**Figure 30:**
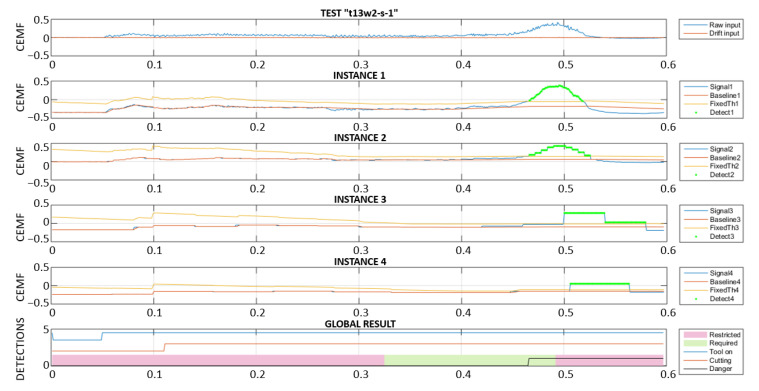
Multi-framerate algorithm: fast approach detection.

In the following two plots (Figure 29 and Figure 30), a slow approach is detected by instance 4, which uses the slowest framerate. The next figure is a fast-approaching event detected in time by instances 1 and 2. The last subplot in both figures includes a “Danger” signal, corresponding to the algorithm output flagging the instant when the tool would have been stopped. For this reason, it remains active after the first activation. Note that, when the algorithm performs properly, the detection needs to happen within the “Required” region, and not within the “Restricted” region of the plot.

### 5.3. Artifacts and Power Consumption

After compensation, significant deviations that do not correspond to any event appear. However, when correlated to reality, these deviations seem to occur mainly when cutting operations encounter any discontinuity. Luckily, such discontinuities tend to be observable through the motor current consumption signal. For this reason, and with the aim of reducing false alarms, the four instances corresponding to different approach speeds will be dynamically adjusted according to the motor power consumption.

**Figure 31:**
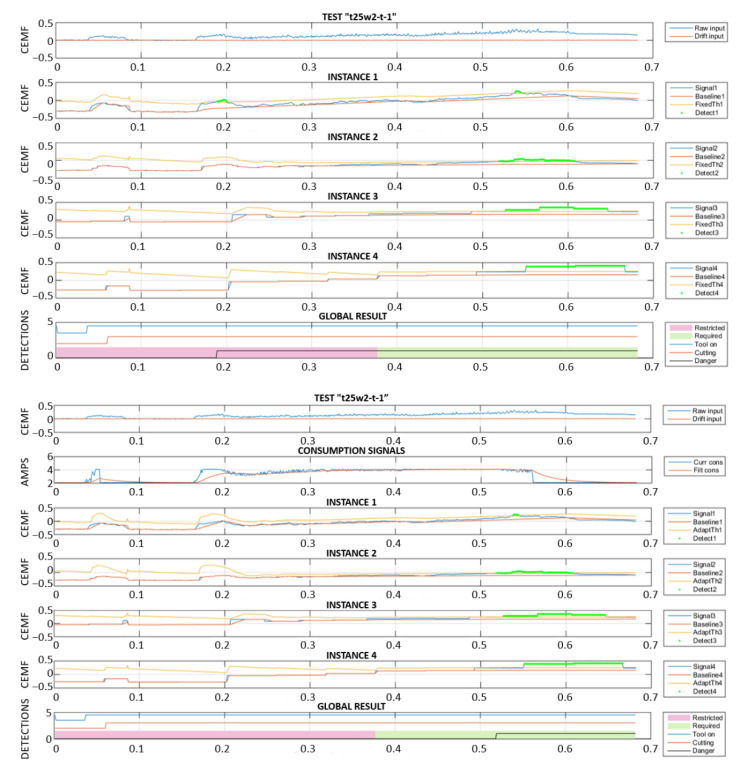
Fixed vs. adaptive algorithm.

Figure 31 shows the results of the two different implementations, without and with adaptive baseline and threshold. The top part shows how a deviation corresponding to a change in the motor load conditions causes a fault trigger in the “Restricted” area. This is a failure since the event must be detected in the “Required” area only. The bottom part of the figure displays the same data, but this time they are processed with an adaptive algorithm that will: (a) absorb disturbances into its baseline faster according to the change in current consumption. (b) Increase the detection threshold according to the change in current consumption.

These two simple but effective changes made it possible to move the detection from the “Restricted” to the “Required” area.

### 5.4. Parameter Optimization

Due to the multiple algorithm instances, the number of parameters of each instance, and the number of situations that these algorithms must cover, it is impractical to tune them by hand. Therefore, a Python script was built to run through all the data with a different configuration set each time and perform a Monte Carlo-like analysis that finds the optimal parameter setting.

**Figure 32:**
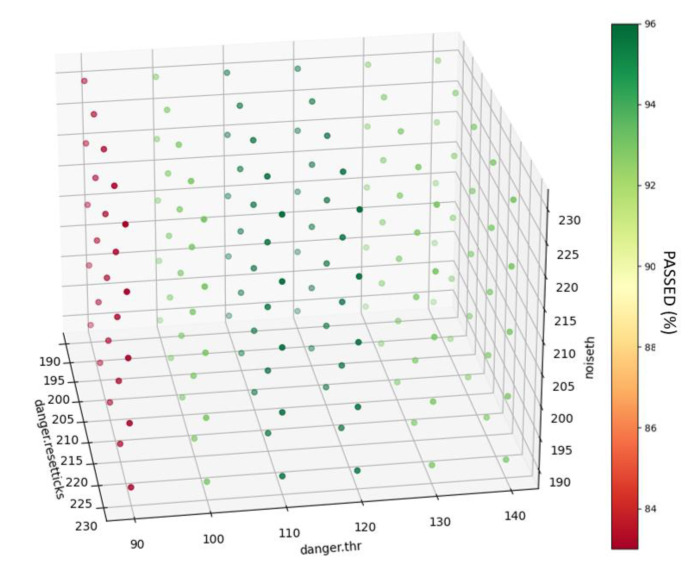
Output of the parameter optimization tool.

Initially, the software generates a random set from the input parameters. The size of the search depends on the number of input parameters and their range. After that, the application runs the algorithm and evaluates the results. Finally, it presents the results and returns the most optimal combination. In Figure 32, a representation of a simple, three-parameter optimization is displayed.

## 6. Validation

**Figure 33:**
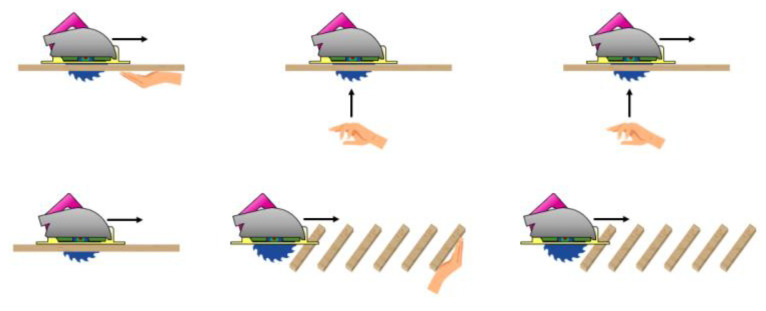
Basic test concepts.

Figure 33 shows where the hand is approaching from and how the wood is distributed across the basic test concepts. A test-dictionary was defined to cover all typical accidents. Then, a test jig was built to make sure all tests would be reproducible in a consistent manner and so the pass/no-pass criteria would equally apply across different tool versions.

The test-dictionary combines the following concepts in a total of 44 different tests:

- Front detection/bottom detection.
- Low speed/high speed cuts.
- Hand not moving/low speed/high speed hand approach.
- Continuous cut/intermittent cut (through beams).
- With/without guide rail.
- Self-protection/third person.
- No fault positives on hard/soft/wet wood.
- Dry/wet wood.
- With/without gloves or safety shoes.

**Figure 34:**
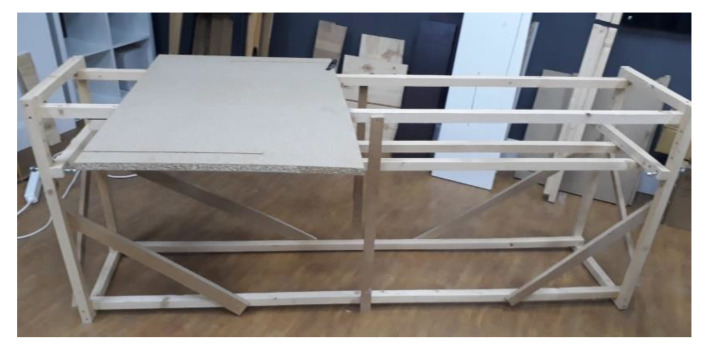
Test jig/workbench.

Figure 34 shows the basic structure of the test jig made out of wood slats secured with screws. This structure was used to fix any other part required to run the test and the piece of wood to work on. It was designed to guarantee a rigid geometry while optimizing weight, cost, and portability.

**Figure 35:**
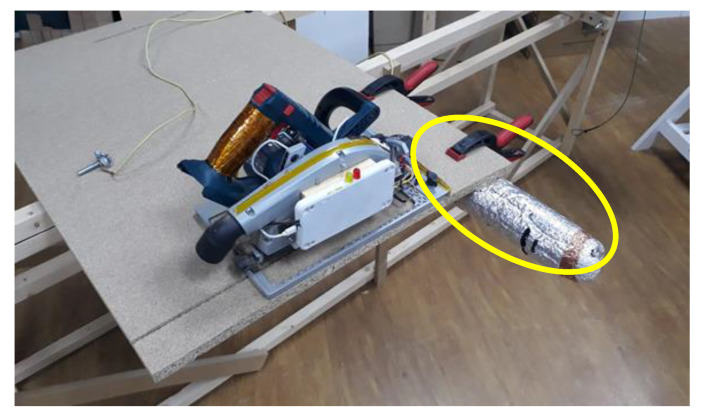
Dummy arm.

As mentioned above, the test jig supported other parts required to run the test. One of the key elements was the dummy arm used to excite the sensor. Figure 35 shows a foam tube covered in metallic paper that acted as a dummy hand (see yellow oval). By connecting it to the user through a simple wire it was possible to explore the full test-matrix without compromising safety.

**Figure 36:**
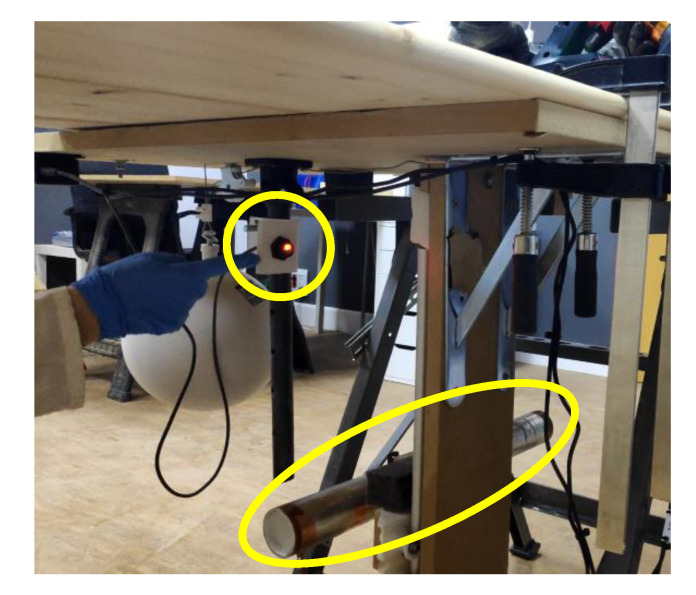
Laser sensor and dummy arm. Bottom detection.

As previously introduced in Figure 26 and now in Figure 36, there are some simple mechanisms with pulleys and weights that help to maintain stable and repetitive speed across different test runs. The basic idea is to multiply the speed of a dropping weight by using pulleys and using this speed to move the dummy arm towards the blade at a repetitive speed. Once the jig was completed, the speed of the arm when crossing the laser beams (see small yellow oval) was characterized. This characterization allowed us to position the dummy arm in the space at the moment of the detection.

**Figure 37:**
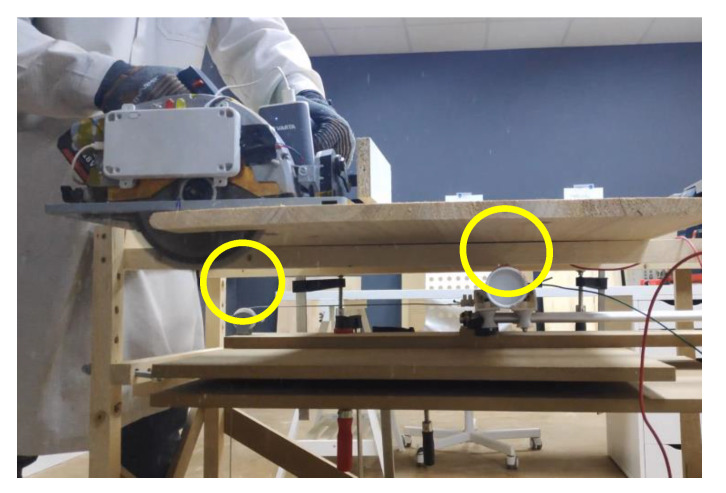
Front detection test.

Figure 37 is an image taken during a front side testing. Appreciate the dummy hand underneath the table indicated in a yellow circle. Appreciate the pulleys and arm in yellow circles now assembled in a vertical configuration.

**Figure 38:**
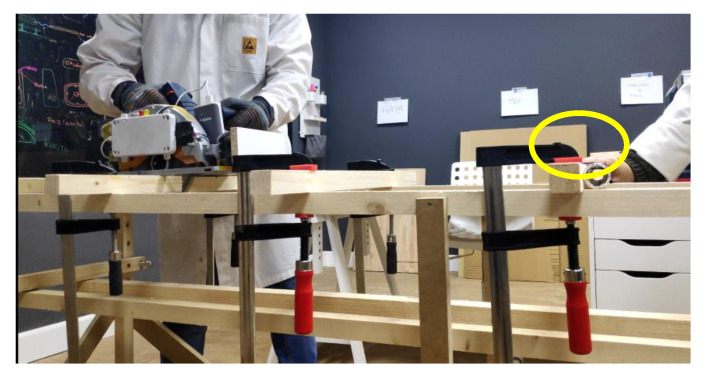
Beam test.

One of the most complicated tests, shown in Figure 38, was the one where several wood beams needed to be cut through with no event detection with the exception of the last beam, where the dummy hand was placed awaiting the blade (yellow oval). The fast transitions of air–wood and wood–air plus the lack of mechanical stability due to the saw not resting on a flat surface make the signal very complicated, making the sensor fail in some test repetitions.

Each test performed has generated data that were recorded together with video data. Through the data, it was possible to determine the detection distance, and the video helped to visually double-check some particular cases.

**Figure 39:**
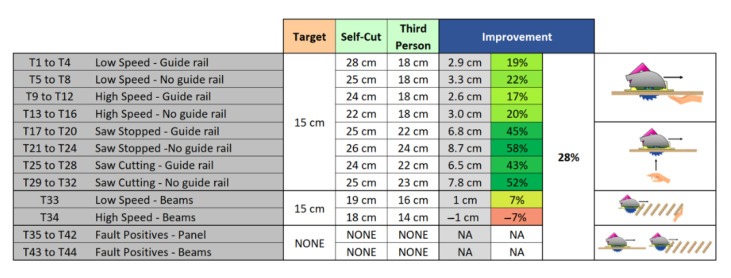
Test results.

As shown in Figure 39, in most of the tests there is a greater detection distance than the target value. Only one block failed to perform as desired, but even though it failed, it was only by 7%. Thus, the results are very close to specifications in all cases. Overall, the power tool delivers an additional 28% average detection distance.

## 7. Conclusions

Through this work, the CEMF technology has been adapted to operate in a very challenging environment where the proposed solution must deal with surrounding grounded metallic pieces, constant strong vibrations, and the uncertainty of the intrinsic random nature of the wood itself. The main benefit and innovation of the proposed solution as opposed to current solutions are that a contactless solution has been developed; meaning that the user does not need to be harmed before being detected, improving the operator mobility and ensuring the safety of both the operator and the rest of the users close to the power tool as has been proved in Section 6. Moreover, the proposed system is capable of stopping the power tool before the user interacts with the disc. In order to perform this action, the system is composed of a CEMF generator, a shield, and firmware that implements a multi-framerate algorithm. These three elements are combined to overcome the challenges of this demanding application that must react equally well to very fast and very slow events.

In order to design and implement the proposed solution, a thorough analysis of how the human presence modifies the electromagnetic field surrounding the tool has been carried out using advanced multi-physics simulators. Thus, it has been proven that the usage of this software is an effective mechanism to study and optimize the generation of the electromagnetic field by maximizing the change at the point of interest. This fact is critical to minimize failures during the test stage.

Additionally, during this work, a methodology to measure the complex impedance of the tool under operating conditions was developed. Note that this parameter is crucial to secure the design of a shield circuitry capable of operating properly in the system. Moreover, in order to assess different algorithm implementations, a virtual testbench has been developed. This virtual environment allowed us to test and improve the whole system without wasting raw materials, such as wood, or energy. It also allows multiple cases to be tested, preventing a person from interacting with the power tool in the early stages of development. Note that this virtual test environment is in continuous development and improvement, and, at this time, the authors are working to integrate the detection of possible failures in the sensor or any related part [21], as well as new algorithms based on interval observers to improve the CEMF algorithm performance [22,23].

Finally, in order to assess the performance of the proposed solution, a testing jig has been developed to be able to run tests in a reliable and feasible manner.

Future research focuses on algorithm improvements to address the issues found in the beam testing, as it is one of the most complicated situations to reliably detect a user and, at the same time, not generate fault triggers. Solving such a problem may require an improvement in terms of sample rate to increase the level of detail required to be able to correctly classify the patterns. Therefore, future work may also be extended from algorithm side to hardware side.

## Data Availability

Not applicable.
